# Metformin attenuates hypothalamic inflammation via downregulation of RIPK1-independent microglial necroptosis in diet-induced obese mice

**DOI:** 10.1038/s41420-021-00732-5

**Published:** 2021-11-08

**Authors:** Xuan Li, You Cai, Jiao Luo, Jingyun Ding, Guojun Yao, Xiaohua Xiao, Yizhe Tang, Zhen Liang

**Affiliations:** 1 Department of Gerontology, Institute of Translational Medicine, The First Affiliated Hospital of Shenzhen University, Shenzhen Second People’s Hospital, Shenzhen, 518035 China; 2 Shenzhen Iinstitute of Advanced Technology, Chinese academy of sciences, Shenzhen, 518035 China; 3 Department of Neurology, Institute of Translational Medicine, First Affiliated Hospital of Shenzhen University, Shenzhen Second People’s Hospital, Shenzhen, 518039 China; 4 Department of Rehabilitation Medicine, Dapeng New District Nan’ao People’s Hospital, the First Affiliated Hospital of Shenzhen University, Shenzhen, 518121 China; 5 Department of Neurology, Shenzhen Institute of Translational Medicine, Guangdong Key Laboratory of Systems Biology and Synthetic Biology for Urogenital Tumors, The First Affiliated Hospital of Shenzhen University Health Science Center, Shenzhen Second People’s Hospital, Shenzhen, 518035 China; 6 Shenzhen People’s Hospital, Shenzhen, 518020 China

**Keywords:** Microglia, Obesity

## Abstract

Necroptosis, a form of programmed cell death, accounts for many inflammations in a wide range of diseases. Diet-induced obesity is manifested by low-grade inflammation in the mediobasal hypothalamus (MBH), and microglia are implicated as critical responsive components for this process. Here, we demonstrate that microglial necroptosis plays a pivotal role in obesity-related hypothalamic inflammation, facilitating proinflammatory cytokine production, such as TNF-α and IL-1β. Treatment with the anti-diabetic drug metformin effectively reduces the obese phenotypes in the high-fat diet (HFD)-fed mice, attributing to remission of hypothalamic inflammation partly through repressing microglial necroptosis. Importantly, using the receptor-interacting protein kinase 1 inhibitor, necrostatin-1s, could not suppress the microglial inflammation nor prevent body weight gain in the obese mice, indicating that the microglial necroptosis is RIPK1-independent. Altogether, these findings offer new insights into hypothalamic inflammation in diet-induced obesity and provide a novel mechanism of action for metformin in obesity treatment.

## Introduction

Diet-induced obesity (DIO) has emerged as a major health problem provoking the interests of researchers worldwide. Many studies have demonstrated that obesity-induced chronic low-grade inflammation in the area of the hypothalamus (mediobasal hypothalamus, MBH), including the arcuate nucleus (ARC), plays a pivotal role in metabolic disorder and body weight gain [1]. Necroptosis, a newly defined well-regulated necrosis, is critical for inflammation regulation. Receptor-interacting protein kinase 1 (RIPK1) and RIPK3 are two key initiators of necroptosis. Their downstream molecule mixed lineage kinase domain-like protein (MLKL) is a critical effector of the pathway. Traditionally, necroptosis initiation requires RIPK1, which binds to its partner RIPK3 to form necrosome. Subsequently, MLKL is recruited and phosphorylated by RIPK3, resulting in cell membrane disruption and release of proinflammatory mediators or damage-associated molecular patterns (DAMPs) [2]. However, increasing evidence has indicated that RIPK3 can mediate RIPK1-independent necroptosis [3]. Functionally, necroptosis is essential for several physiological and pathological processes, including neuroinflammation. Previous studies have found necroptosis activation in retinal degeneration and Alzheimer’s disease [4, 5]. Therefore, it is noteworthy to examine the functional roles of necroptosis in hypothalamic inflammation in DIO.

Microglia are the resident macrophage population in the central nervous system (CNS). In DIO, proinflammatory cytokine production in the MBH, including TNF-α and IL-1β, coincides with hypothalamic microglial activation and accumulation [6]. Therefore, it is not surprising to consider microglia as critical sensors that orchestrate the metabolic inflammation in the MBH [7].

Metformin is first-line therapy for type 2 diabetes (T2DM). Extensive cohort studies have shown body weight loss associated with metformin treatment [8]. Emerging evidence suggests that metformin can repress hepatic inflammation in fatty liver disease. Nevertheless, whether metformin regulates metabolic inflammation in the MBH remains uncertain.

In this study, we identified that microglial necroptosis induces metabolic inflammation in DIO mice. Metformin suppressed the microglial necroptosis and subsequent inflammation. Our work unravels the link between necroptosis and hypothalamic inflammation, argues the therapeutic potential of metformin on hypothalamic inflammation in DIO mice, and dissects its possible mechanism in vivo and in vitro.

## Results

### RNA-Seq analysis reveals necroptosis arises in the MBH of DIO mice, and metformin treatment dampens this process

**Fig. 1:**
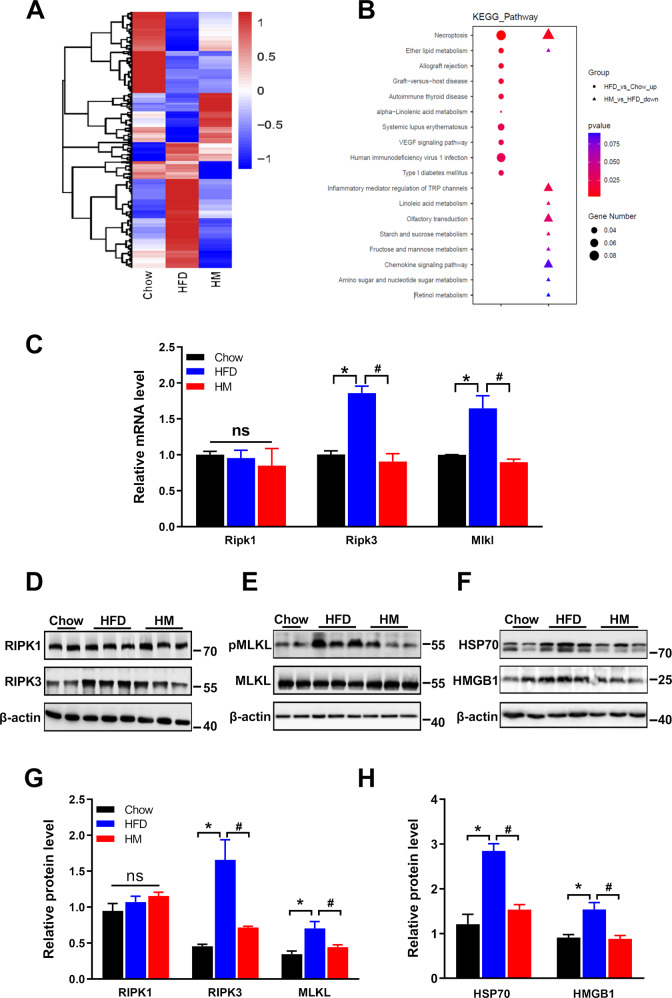
Necroptosis occurred in the hypothalamus of DIO mice, and metformin suppresses the process of necroptosis. **A** Heat map of RNA-Seq data showed changes of hypothalamic genes in Chow (*n* = 4), HFD (*n* = 4), and metformin-treated mice (300 mg/kg/day for six weeks, HM, *n* = 4). **B** Advanced bubble chart revealed the enrichment of differentially expressed genes in signaling pathways. **C** The mRNA levels of necroptosis biomarkers, *Ripk1, Ripk3*, and *Mlkl*, in the hypothalamus of three groups of mice, are measured by qPCR with independent RNA samples. Increased expression levels of *Ripk3* and *Mlkl* were observed in HFD. Metformin dampened the overexpression of the genes. There was no detectable difference in *Ripk1*. **D**, **E** and **G** Immunoblotting data confirmed the findings in qPCR (*n* = 7 for each group of mice). **F** and **H** The upregulated protein expression levels of HSP70 and HMGB1, two DAMPs, validated the necroptosis occurrence in the hypothalamus of obese mice and metformin mitigation of the injury. The data were presented as mean ± SEM. *P < 0.05 vs. Chow mice; ^#^*P* < 0.05 vs. HFD mice.

To investigate potentially altered gene expression underlying the DIO mice, RNA-sequence (Seq) analysis was performed on the hypothalamus of the chow mice (Chow), high-fat feeding mice (HFD), and the metformin-treated HFD mice (HM) (Fig. 1A). Differential expression (DE) analysis identified 765 DE genes in HFD compared with that in Chow, consisting of 319 upregulated and 446 downregulated genes (Supplementary Table 1). Meanwhile, 246 upregulated and 349 downregulated total in 595 genes were obtained in HM compared with that in HFD (Supplementary Table 2). KEGG enrichment analysis indicated that necroptosis positioned in the overlap of the two clusters of HFD_up versus Chow and HM_down versus HFD (Fig. 1B). Of note, as the most required component for necroptosis, the mRNA expression of *Mlkl* was found to be remarkably upregulated in the HFD hypothalamus compared with that in Chow (log2foldchange = 1.26, *p* = 0.046), while being significantly downregulated in the HM vs. HFD (log2foldchange = −1.58, *p* = 0.047).

To confirm the RNA-Seq results, we first examined the mRNA expression level of *Ripk1* and *Ripk3*, and *Mlkl* utilizing quantitative polymerase chain reaction (qPCR) analysis with independent RNA samples. As shown in Fig. 1C, the expression pattern of *Mlkl* was alike with RNA-Seq data. However, the expression of *Ripk3* exhibited a moderate increase in the hypothalamus of the HFD mice but a significant decrease in HM mice, which were not observed in RNA-Seq data. No significant differences were found in the gene expression of *Ripk1* among the three groups, consistent with the findings in RNA-seq. We next determined the protein expression level of RIPK1, RIPK3, and MLKL by immunoblotting. The results revealed that the level of RIPK3 expression and MLKL expression were upregulated in the HFD mice, while metformin adjusted the abnormality. Likewise, no significant difference was found in the expression of RIPK1 among the three groups (Fig. 1D, E and G).

To further illustrate necroptosis-mediated hypothalamic injury, we explored high-mobility group box-1 (HMGB1) expression, the major DAMP released from necroptotic cells [9]. Of note, HFD feeding reinforced HMGB1 expression, whereas metformin suppressed the protein nearly to the baseline level. Similarly, heat shock protein 70 (HSP70), a stress-inducible heat shock protein promoting MLKL polymerization [2], was boosted in the HFD-fed mice and attenuated in the metformin-treated mice (Fig. 1F and H). Overall, HFD feeding induced necroptosis in the hypothalamus, and metformin mitigated the process.

### Metformin attenuates hypothalamic inflammation by suppressing the production of proinflammatory cytokines, TNF-α and IL-1β, and protecting POMC neurons from injury

**Fig. 2:**
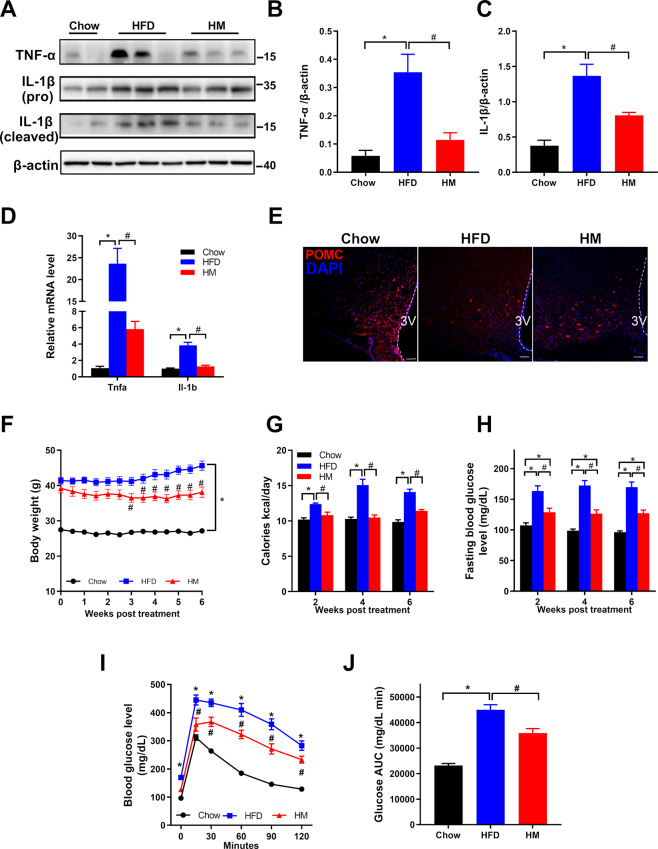
Metformin treatment attenuated hypothalamic inflammation, leading to body weight loss and glucose homeostasis improvement in DIO mice. **A**–**C** Immunoblotting data showed elevated expression of TNF-α and IL-1β in the hypothalamus from HFD mice. The overexpression of these proinflammatory cytokines in the hypothalamus was declined in HM mice. **D** qPCR results that aligned with the data in immunoblotting. **E** Immunostaining for POMC cells (red) in the MBH (*n* = 4) showed metformin protection of POMC cells from HFD-induced injury. Scale bar represented 50 μm. **F**–**J** General index demonstrated the beneficial effects of metformin on obese mice. **F** and **G** Metformin lowered the body weight significantly and the caloric intake in HM mice compared with those in HFD mice. **H** Metformin downregulated fasting blood glucose concentration in DIO mice. **I** Glucose tolerance test (GTT, glucose 1 g/kg intraperitoneally) in mice fasted for 12–16 h. **J** Areas under the curve (AUC) for GTT (*n* = 7). The data were presented as mean ± SEM. *P < 0.05 vs. Chow mice; ^#^*P* < 0.05 vs. HFD mice.

The release of proinflammatory cytokines in the hypothalamus, such as TNF-α and IL-1β, is a hallmark of chronic HFD feeding. To evaluate the effect of metformin on these inflammatory mediators, immunoblotting and qPCR were both employed. Results showed that HFD triggered higher tumor necrosis factor-alpha (TNF-α) and interleukin 1-beta (IL-1β) expression than the chow diet. In contrast, metformin treatment significantly downregulated the cytokines’ expression markedly both on the protein level and the mRNA level (Fig. 2A–D). POMC neurons, located in the hypothalamus, respond to metabolic cues to control food intake and energy expenditure. Immunofluorescence staining demonstrated that HFD mice obsessed fewer POMC-expression neurons in the ARC than their chow rivals. Indeed, metformin prevented further repression of POMC neurons in the MBH from HM mice (Fig. 2E).

Next, we examined the effects of metformin on general indicators of obese mice. As we expected, HFD mice exhibited a striking increase in body weight gain and food intake than the Chow group. Nevertheless, HM mice weighed significantly less than the HFD group from the third week and continued to weigh less throughout the experiment for six weeks (Fig. 2F). Besides, metformin administration reversed HFD-induced elevation of caloric intake compared to that in the HFD mice receiving vehicle treatment (Fig. 2G). Also, metformin improved the glucose homeostasis of the HFD mice even before the weight loss. After two weeks of metformin treatment, the HM mice began to show lower fasting blood glucose levels (HM 128.8 ± 6.5 vs. HFD 163.3 ± 8.6 mg/dL; Fig. 2H) and a significant improvement in glucose tolerance (Fig. 2I and J), showing the calculated area under the curve. We observed that metformin could improve glucose homeostasis from two weeks to the end of the experiment. These results aligned with the notion that metformin moderately decreased weight gain caused by a high-fat diet [10, 11]. These findings collectively suggested that metformin promoted body weight loss, reduced food intake, and rescued the impaired glucose metabolism in DIO mice by suppressing metabolic inflammation in the MBH.

### Metformin inhibits microglial necroptosis in the hypothalamus of DIO mice

**Fig. 3:**
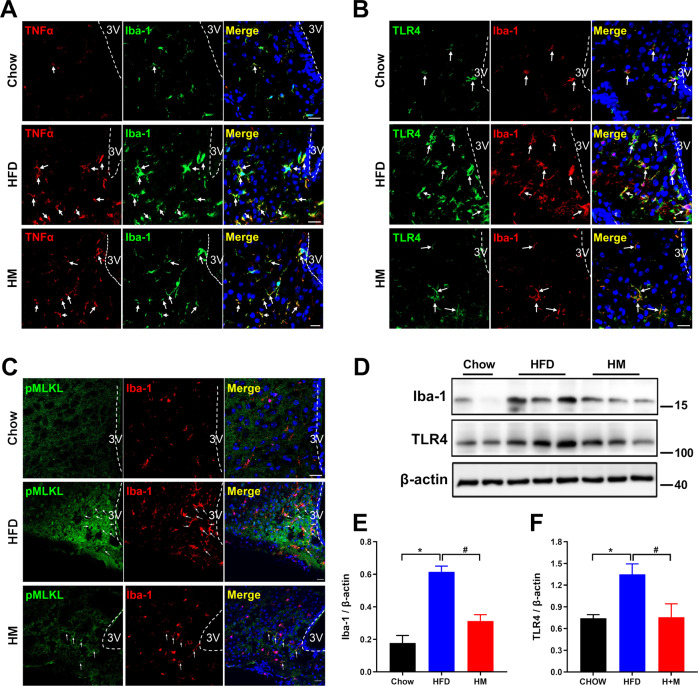
Metformin reduced microgliosis by suppressing necroptotic microglia in the MBH of DIO mice. **A** Immunostaining for TNF-α (red) and Iba-1 (green, a marker of activated microglia) in the MBH showed that chronic HFD feeding induced Iba-1 positive microglia accumulation and strong TNF-α immunoreactivity within the area of microglia. Metformin remarkedly decrease the microgliosis and TNF-α positive staining overlapped with Iba-1, indicating metformin downregulated microglial inflammation effectively. **B** Immunofluorescent staining for TLR4 (green) and Iba-1 (red) in the MBH indicated that TLR4 expression was observed on the surface of Iba-1 positive microglia in HFD mice and that HM mice exerted a reduced amount of microglia and corresponding decreased TLR4 staining. **C** Immunofluorescent double-staining for p-MLKL (green) and Iba-1 (red) in the MBH. Iba-1 immunoreactivity was co-localized with pMLKL immunoreactivity, demonstrating microglia underwent necroptosis caused by chronic HFD feeding. Metformin treatment markedly repressed microglial necroptosis in HM mice (*n* = 4). White arrows indicated the merge of the two colors. Scale bar represented 20 μm. **D**, **E**, **F** Immunoblotting data for Iba-1 and TLR4 expression. The data were presented as mean ± SEM. *P < 0.05 vs. Chow mice; ^#^*P* < 0.05 vs. HFD mice.

As activated microglia are considered the primary source of proinflammatory cytokine and the critical mediator of inflammation response during hypothalamic inflammation, we first figured out whether metformin affected the microglia by comparing Iba-1 positive microglial cells in the ARC region of three groups of mice using immunostaining. Mice consuming HFD accumulated more Iba-1 positive cells in the ARC region (microgliosis) (Fig. 3A). These cells were observed amoeboid or round shape, representing activated microglia morphology. Intriguingly, metformin administration decreased the number of Iba-1 positive microglia and modified these cells’ shape showing longer branching processes and smaller cellular bodies. Furthermore, We also measured hypothalamic inflammation in this setting by staining for TNF-α. TNF-α expression was elevated in MBH of HFD mice and strongly co-localized with Iba-1 positive microglia (Fig. 3A). In contrast, mice receiving metformin exhibited a lower level of TNF-α and fewer double-positive cells.

Next, we detected the expression of TLR4 in Iba-1 positive microglia since TLR4 is a critical mediator for hypothalamic inflammation. As shown in Fig. 3B, TLR4 immunoreactivity has a strongly positive correlation with Iba-1 expression: TLR4 was predominantly double-stained with Iba-1; the expression level of TLR4 increased in HFD while decreased in metformin-treated mice. Alternatively, immunoblotting was performed for further confirmation. Iba-1 expression was boosted in the HFD group compared with Chow mice but declined in the HM group. The same expression pattern was also shown in the TLR4 expression (Fig. 3D–F). As TLR4 is also responsible for inducing the necroptotic cascade reaction, it is not surprising to detect the expression of MLKL in hypothalamic microglia. Stronger positive immunoreactivity for pMLKL was observed in the HFD group, which partially overlapped with the areas of Iba-1-positive cells. In contrast, the intensity and the expression of pMLKL were restored after metformin treatment, indicating that metformin dampened obesity-induced hypothalamic inflammation partly, if not all, via regulating necroptotic microglia (Fig. 3C). Together, these findings demonstrated that metformin suppressed the microglial necroptosis and subsequent production of TNF-α and IL-1β, resulting in improving metabolic inflammation in the MBH.

### Necroptosis blockade using Nec-1s shows no effect on lipopolysaccharide stimulation in BV2 cells in vitro

**Fig. 4:**
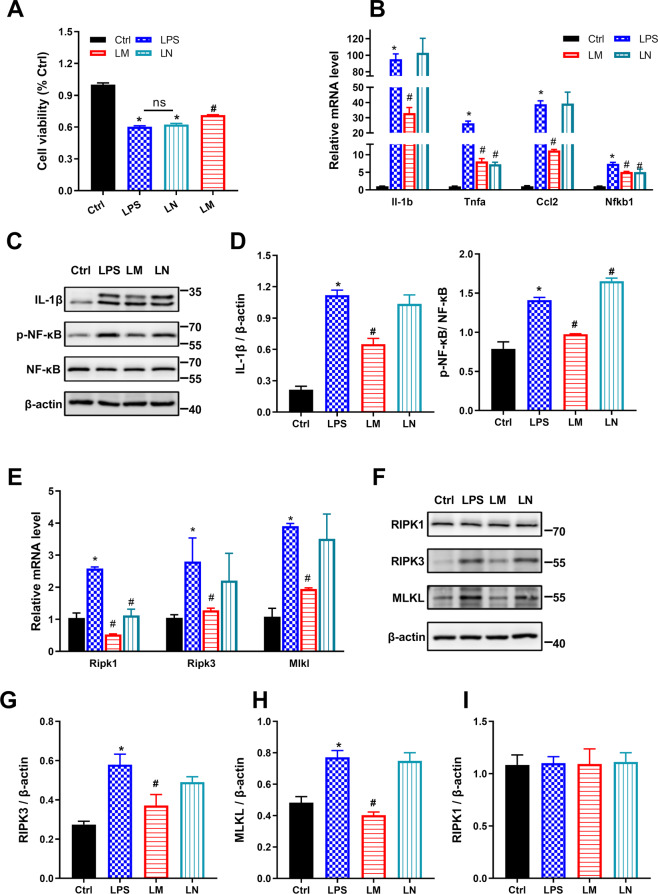
Necroptosis blockade using Nec-1s shows no effect on LPS stimulation in BV2 microglial cells in vitro. Cultured BV2 microglial cells were stimulated with LPS (500 ng/ml) for 12 h before adding LPS, metformin (2.5 mM), and Nec-1s (0.5 μM) for an additional 12 h, respectively. **A** Cell viability analysis showed ~40% cell death in LPS cells and ~72% cell survival in the LM group. No significant difference was evident after Nec-1s adding compared with LPS. **B** qPCR data showed proinflammatory genes were upregulated by LPS, whereas downregulated by metformin. There was no difference observed in Il-1b and Tnfa gene expression after Nec-1s treatment in BV2 cells. Nevertheless, the mRNA of Tnfa and Nfkb1 were expressed at lower levels in the LN cells. **C**, **D** Immunoblotting data showed lower expression levels of IL-1β and NF-κB in the LM group, indicating metformin anti-inflammation effects on LPS-stimulated cells. Nec-1s adding caused slightly increased NF-κB activation but similar IL-1β expression with that in the LPS group. **E**–**I** qPCR and immunoblotting for RIPK1, RIPK3, and MLKL. The expressions of RIPK3 and MLKL were elevated in LPS and LN but reduced in LM. RIPK1 was upregulated in LPS and downregulated by LM and LM at the mRNA level, but no difference was observed in the four groups, implying LPS-induced necroptosis in BV2 cells were RIPK1-independent. *n* = 3 independent cultures. The data were presented as mean ± SEM. *P < 0.05 vs. Control cells; ^#^*P* < 0.05 vs. LPS-stimulated cells.

To further determine the effects of metformin on microglial necroptotic inflammation in vitro, murine BV2 microglial cells were cultured and treated with lipopolysaccharide (LPS), which mimicked the HFD-induced microglia activation in the hypothalamus. First, we examined the cell viability of BV2 cells after treatments. LPS stimulation induced ~40% cell viability down to the control. More cells were rescued after being treated with metformin (~72%) (Fig. 4A). Next, we measured the mRNA expression of Il-1b, Tnfa, C-C motif chemokine ligand 2 (Ccl2), and nuclear factor-kappa B subunit 1 (Nfkb1) in BV2 cells utilizing qPCR analysis. As shown in Fig. 4B, the expression levels of these genes were dramatically elevated in LPS-treated BV2 cells (the LPS group) compared with the control cells (the Ctrl group). By contrast, these expressions’ elevation was reduced over 50% by metformin (the LM group). Similarly, the protein expression levels of IL-1β and phospho-NF-κB (p-NF-κB) were also upregulated by LPS and downregulated by metformin (Fig. 4C and D). Nec-1s, a selective RIPK1 inhibitor, was applied to determine necroptosis involvement in microglial inflammation. Notably, adding Nec-1s alone after LPS stimulation (the LN group) did not improve the cell injury caused by LPS (Fig. 4A). The mRNA expression of Il-1b and Ccl2 of the cells was comparable with the LPS group. However, the mRNA of Tnfa and Nfkb1 were expressed at lower levels in the LN cells than that in the LPS cells (Fig. 4B). Surprisingly, immunoblotting data suggested an opposite expression trend at protein levels: IL-1β expression in the LN group was comparable with that in the LPS group; NF-κB activation was even slightly enhanced in LN cells in contrast to LPS cells.

We then turned to access the necroptosis profile of BV2 microglial cells. It could be seen that the LM group expressed significantly less RIPK3 and MLKL than the LPS group both at mRNA and protein levels and that no significant differences were found between the LPS group and the LN group (Fig. 4E–H). Although *Ripk1* mRNA was increased over twofold induced by LPS and declined significantly both in metformin-treated and Nec-1s-treated cells (Fig. 4E), the RIPK1 protein expression was comparable in all groups (Fig. 4I).

### Combined use of metformin and Nec-1s does not enhance the effect of metformin alone in vitro

**Fig. 5:**
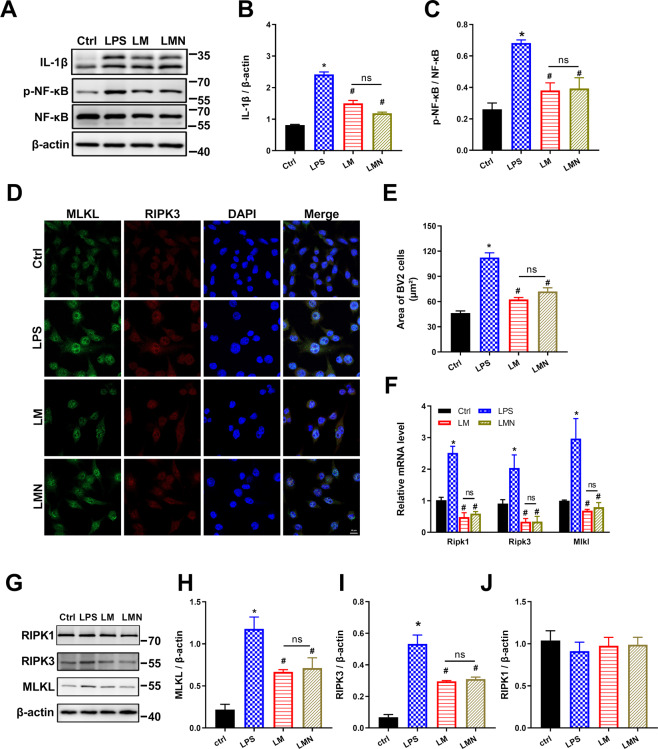
Combined use of metformin and Nec-1s does not enhance the effect of metformin alone in vitro. **A**–**C** Immunoblotting for IL-1β and NF-κB signal pathway. LMN treatment reduced the expression of the inflammation mediators, although no difference greater than metformin alone was observed. **D** Immunofluorescent staining for MLKL and RIPK3 in BV2 cells. Weak signals for MLKL protein (green) were diffusely distributed around the nuclei in the control group. Accumulation of positive immunoreactivities in the cytoplasm surrounding the nuclei was seen in LPS. Fewer spot-like fluorescent signals were detected in the zone in LM and LMN. Positive signals for RIPK3 (red) were also seen in the same expression manner as MLKL. The signals were overlapped in part. **E** The graph showed enlarged cell size in LPS-stimulated cells, which turned smaller in LM cells and LMN cells. **F** Necroptosis-related gene expression was measured by qPCR. No significant difference was found in LM and LMN. **G**–**J** Immunoblotting for necroptosis-related proteins. Except for no effect on RIPK1, the combination of metformin and Nec-1s downregulated the protein mentioned above. However, the effects seemed similar to metformin. *n* = 3 independent cultures. Five images were collected randomly in each culture, and the experiments were repeated at least three times. Scale bar=10 μm. The data were presented as mean ± SEM. *P < 0.05 vs. Control cells; ^#^*P* < 0.05 vs. LPS-stimulated cells.

To assess the combined action of metformin and Nec-1s, first, we examined the expression of IL-1β, NF-κB by immunoblotting. The cells in LMN(the metformin-Nec-1s combination group) exert a decreased level of the proteins compared with LPS-treated cells, although no difference greater than the LM cells was observed (Fig. 5A–C). Then, we compared their effects on MLKL distribution and expression utilizing immunofluorescence and immunoblotting. We observed the enlarged nuclei with a round shape in LPS-treated cells, whereas the cells in LM and LMN were partly spindle-like and exhibited smaller sizes on average (Fig. 5D and E). Weak signals for MLKL protein were diffusely distributed around the nuclei in the control group. LPS treatment led to an accumulation of positive immunoreactivities in the cytoplasm surrounding the nuclei.

In contrast, fewer spot-like fluorescent signals were detected in the zone after metformin treatment, which was observed as well in the LMN group. Meanwhile, positive signals for RIPK3 protein were also seen in a similar pattern as MLKL. Remarkably, the signals of the two proteins were overlapped in part. Data in Fig. 5F and G–I confirmed the findings of MLKL and RIPK3 above both at mRNA level and protein level. Also, RIPK1 expression was comparable within all the groups (Fig. 5J). Collectively, exposing BV2 microglial cells to LPS induced necroptotic inflammation, which was rescued by metformin; no clear benefit of Nec-1s alone or combination with metformin could be identified within the analysis, indicating that inhibiting RIPK1 may not be involved in the process of LPS-induced necroptosis in BV2 microglial cells.

### HFD-induced necroptosis in the MBH is RIPK-1 independent in vivo

**Fig. 6:**
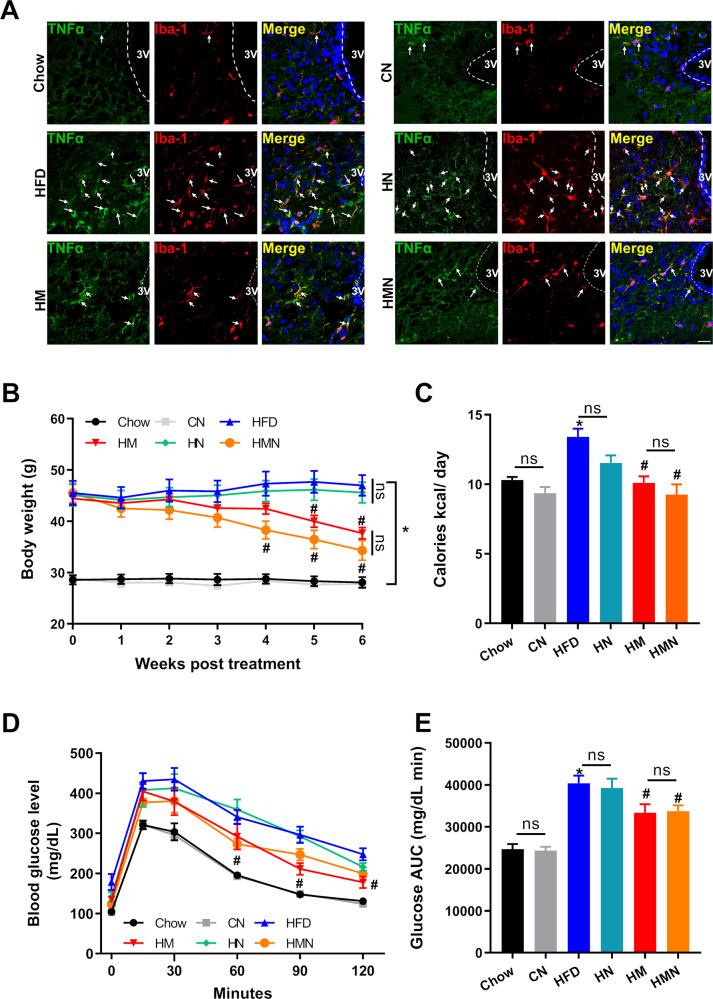
Microglial necroptosis in the hypothalamus was RIPK1-independent in DIO mice. DIO mice were divided into six groups: Chow, CN (chow diet+ Nec-1s 2.5 mg/kg intraperitoneally), HFD, HN (HFDt+ Nec-1), HM (HFD+ metformin 300 mg/kg, oral gavage), and HMN (HFD+ metformin+ Nec-1). **A** Immunostaining for TNF-α (green) and Iba-1 (red) in the MBH from six groups of mice (*n* = 5). The two proteins’ signals in Chow, HFD, and HM were similar to those described in Fig. 3A. The positive signals of TNF-α and Iba-1 were comparable in the hypothalamus between Chow and CN. A high number of double-stained Iba-1 and TNF-α cells was observed in HN, similar to those in HFD, indicating RIPK1 blockade did not influence hypothalamic inflammation. Additionally, semblable observations for the two proteins were obtained from HM mice and HMN mice. Five images were acquired for each group. Scale bar=20 μm. **B** and **C** Body weight gain and caloric intake of mice from the six groups. **D** and **E** GTT and the AUC data from the mice of different treatments. The data were presented as mean ± SEM. *P < 0.05 vs. Chow mice; ^#^*P* < 0.05 vs. HFD mice.

To validate the notion that DIO-mediated microglial necroptosis was RIPK1-independent, we built up the obese mice model receiving the treatments of metformin, necrostatin-1 (Nec-1, HN), and metformin plus Nec-1 (HMN). Chow mice were also administrated with Nec-1 (CN) as control. As shown in Fig. 6A, the Nec-1 treatment did not affect the hypothalamic inflammation of chow-fed mice, as the positive signals of TNF-α and Iba-1 were comparable in the hypothalamus between the groups (Chow and CN). Elevated double-staining of Iba-1 and TNF-α cells was observed both in the hypothalamus from HFD and HN, indicating RIPK1 blockade did not influence HFD-induced hypothalamic inflammation. Significantly, less activated Iba-1 positive microglia overlapped with TNF-α staining were found in the hypothalamus of HM and HMN, and no significant difference between HM and HMN was identified. Body weight analysis did not show any significant difference between the HFD and HN groups (HFD 47.0 ± 2.0 g vs. HN 45.6 ± 2.0 g, Fig. 6B). Although HM mice and HMN mice reduced body weight compared with HFD mice, a clear benefit of adding Nec-1 plus metformin could not be identified in this analysis (Fig. 6B). Caloric intake analysis and GTT test showed similar results (Fig. 6C-E). All data together indicated that HFD feeding induced necroptotic inflammation in the hypothalamus in a RIPK1-independent manner; metformin rescued the necroptotic microglia without affecting the RIPK1 signal.

## Discussion

In the present study, we first delineate a novel picture that microglial necroptosis facilitated metabolic inflammation in the MBH of DIO mice. Next, we provide evidence that metformin inhibited proinflammatory cytokines and microgliosis partially by suppressing the necroptosis process of microglia in vivo and in vitro, which would be a new explanation of its effects on reducing body weight. Another important finding was that neither Nec-1s alone could shunt the microglial necroptosis in vivo and in vitro, nor could combined use of metformin and Nec-1s enhance the effects of metformin alone.

Inflammatory mediators released from microglia result in the long-lasting impaired metabolic control of the hypothalamus [7, 12]. Traditionally, activation of the NF-κB signal transduction pathway was considered critical for proinflammatory cytokines overproduction, including TNF-α, IL-1β, and CCL2 [13, 14]. However, emerging studies have demonstrated that necroptotic microglia contributes to neuroinflammation in several neurodegeneration diseases [15–19]. As microglia locates at the vital position in hypothalamic inflammation, the possibility rises that necroptosis occurs in microglia during the procession of inflammation in the MBH. In this study, we provide evidence that hypothalamic microglia underwent necroptosis in response to chronic HFD stimulation, leading to the exacerbation of cytokine production. Indeed, immunofluorescence showed strong signals of MLKL overlapped with Iba-1 positive cells in HFD mice (Fig. 3C). In vitro analysis validated BV2 microglial cells overexpression of RIPK3 and MLKL at both mRNA and protein levels in response to LPS stimulation. Furthermore, detection of necroptosis-related DAMPs, such as HSP70 and HMGB1 [2, 20], confirmed the occurrence of necroptosis in the MBH in obese mice. Therefore, our data indicate activated microglial were partly inclined to undergo necroptosis, a novel contributor to diet-induced hypothalamic inflammation.

Metformin is commonly used to treat obese-associated T2DM [11]. Prior studies have shown that T2DM subjects treated with metformin obsessed fewer hypothalamic microglia than T2DM subjects without metformin treatment [21]. Meanwhile, animal studies have noted that metformin inhibited neuroinflammation and showed a protective effect [22, 23]. However, the effects of metformin on the microglial necroptosis in the hypothalamus in obesity remain unclear. We observed the recovery of POMC neurons in the MBH after metformin treatment, accounting for the decreased food intake compared with HFD mice, which was in line with the findings before [24]. Markedly, metformin exerted the ability to downregulate necroptosis biomarkers, such as MLKL expressed within hypothalamic microglia. Together, these effects gave rise to the sharp fall of TNF-α expression collocated within Iba-1 positive microglia (Fig. 3A). TLR4 has been found to express abundantly on the surface of microglia during inflammation. It is well known that TLR4 activation is essential for necroptotic signaling [25, 26]. Regarding this, one possible explanation for metformin’s action might be that metformin mitigated the accumulation of activated microglia in the MBH, followed by the corresponding decrease of TLR4 expression (Fig. 3B, D, F). One of the issues that emerged from these findings is the underlying mechanism of how metformin restrained microgliosis. We first considered necroptosis-related microglial death. Nevertheless, it is conflictive to our data that metformin decreased the expression of DAMPs significantly since necroptosis, like necrosis, leads to the release of those inflammatory mediators [9] (Fig. 1F). Besides, cell viability analysis revealed that metformin protected BV2 microglial cells from death caused by LPS in vitro (Fig. 4A). Given that metformin promotes the M1-to-M2 polarization of macrophages in obese adipose tissue [27], we could not rule out the possibility that metformin modulates the M1/M2 polarization of microglia in the MBH, which requires further investigation.

Generally, RIPK1 is considered the initiator of necroptosis due to its kinase activity in RIPK1-dependent necroptosis signaling. On the other hand, emerging evidence also demonstrates that RIPK3 mediates necroptosis in a RIPK1-independent manner, where RIPK1 exerts its scaffold function to promote the NF-κB pathway [3, 28, 29]. In the present study, RIPK protein expression was comparable in all groups in vivo and in vitro, although mRNA expression patterns differed. A possible explanation for this discordance might be that in vivo data reflected the impact of HFD feeding on the entire hypothalamus, including neurons and glia, whereas BV2 microglial cells represent microglia alone. Nevertheless, the poor correlation between mRNA and protein expression levels in vitro requires further investigation to uncover the role of RIPK1 in the microglia. Notably, adding Nec-1s did not affect microglial inflammation. These data point to the possibility that HFD-induced necroptotic inflammation is RIPK1-independent. Nec-1s is the specific kinase inhibitor of RIPK1 and can not affect the scaffold activity of RIPK1, which may explain why the solid TNF-α expression overlapped with Iba-1 positive microglia in the MBH in the HN mice (Fig. 6A). Additionally, data that combined treatment of Nec-1s and metformin exhibited comparable effects with metformin alone further support the idea that the non-catalytic, scaffold activity of RIPK1 facilitates the energy-sensing signaling [30].

There are some questions that need to be addressed. First, our study could not clearly identify why necroptosis happens in the microglia. Prior studies have shown SFAs induce microgliosis and trigger TLR4/IKKβ/NF-κB pathways, resulting in the impaired metabolic disorder of the hypothalamus [12]. Necroptosis is another form of programmed cell death and works as a backup system when apoptosis is blunt [31]. These facts raise the possibility that microglial necroptosis may exhibit a protective effect on microgliosis, regardless of proinflammatory cytokines release. Therefore, it is worthy to distinguish the role of microglial necroptosis in hypothalamic inflammation. Second, our data suggest RIPK1 displays scaffold activities in microglial necroptosis. Further research needs to be carried out to validate the mechanism of RIPK3 and MLKL that they are involved in in the microglial necroptosis. Besides, how metformin initiates the regulation on RIPK3 and MLKL in the process is not well defined in this study.

In conclusion, our study demonstrates that the RIPK1-independent microglial necroptosis is involved in the HFD-induced metabolic inflammation in the MBH, while metformin attenuates hypothalamic inflammation via repressing microgliosis and suppressing the microglial necroptosis. Overall, this study delineates a clearer picture of the hypothalamic inflammation in obesity and provides more evidence for the usage of metformin in obesity-related treatment.

## Materials and methods

### Animal study

C57BL/6 mice were purchased from the Model animal research center of Nanjing University Nanjing, China, and bred under a standard condition of care (specific pathogen-free environment; 20-22 °C; 12:12-h light-dark cycle). The experimental procedures were approved by the Animal Ethics Committee at Shenzhen University, China.

5-week-old male mice were fed a 60% high-fat diet (HFD; D12492, Research Diets, Research Diets, New Brunswick, NJ), with normal-chow diet mice as controls (Chow). After 12 weeks, mice were randomly grouped for different treatments according to the random number table. Firstly, three groups were set, including the Chow mice, HFD control group (HFD, *n* = 15), and metformin administration group (HM, *n* = 15). The drug (300 mg/kg body weight, D150959, Sigma-Aldrich) or vehicle was administrated by oral gavage daily for six weeks. Body weight was recorded twice a week, and food intake was measured every day during the experiment.

In the second batch, necrostatin-1 (Nec-1, S8037, Selleck) was applied in the chow mice and HFD mice at 2.5 mg/kg body weight by intraperitoneal injection daily for six weeks.

### Intraperitoneal glucose tolerance test (GTT)

Intraperitoneal GTT was performed every two weeks after drug administration on 16 h-fasted mice. 1 g/kg body weight glucose was injected intraperitoneally. Blood glucose was measured using a handheld glucometer (OneTouch Ultra Easy, LifeScan) via tail bleed at 0, 15, 30, 60, 90, and 120 min after glucose administration.

### RNA sequencing and bioinformatics analysis

Total RNA was isolated from the hypothalamus of the Chow group (*n* = 3), the HFD group (*n* = 4), and the HM group (*n* = 4) using TRIzol Reagent (15596026, Invitrogen). The following steps were performed at Novogene Bioinformatics Technology Co., Ltd. Briefly, the integrity of RNA was evaluated using the RNA Nano 600 Assay Kit of the Bioanalyzer 2100 system (Agilent Technologies, CA, USA). Sequencing libraries were generated using NEBNext UltraTM RNA Library Prep Kit for Illumina (NEB, USA) following manufacturers’ instructions. Sequencing was performed on an Illumina Novaseq platform and 150-bp paired-end reads were generated. The clean data that passed through the quality control were obtained from the raw data (Table 1) and were subsequently analyzed with methods listed in Table 2. Differentially expressed genes (DEGs) were determined by fold change >2 and a *P* < 0.05. The significance of enriched Gene Ontology (GO) terms was set at less than 0.05.

**Table 1:** Basic information of the sequenced sample data.

Sample	Clean_reads	Error_rate	Q20	Total_map
Chow01	70158630	0.03	97.78	68137262 (97.12%)
Chow02	74377652	0.02	98.07	72430188 (97.38%)
Chow03	69450488	0.03	97.89	67607592 (97.35%)
HFD01	70772642	0.03	97.96	68879724 (97.33%)
HFD02	69488398	0.02	98.08	67661496 (97.37%)
HFD03	72155654	0.03	97.97	70234152 (97.34%)
HFD04	69201722	0.02	98.06	67393277 (97.39%)
HM01	68338818	0.03	97.98	66540152 (97.37%)
HM02	70487014	0.03	97.96	68618258 (97.35%)
HM03	73747200	0.02	98.04	71791775 (97.35%)
HM04	69533272	0.03	97.94	67664643 (97.31%)

**Table 2:** Software used in the clean data analyses.

Analysis	Software	Version
Mapping Analysis	Hisat2	2.0.5
Quantification	FeatureCounts	1.5.0-p3
Differential Expression Analysis	DESeq2	1.16.1
GO and KEGG Enrichment Analysis	clusterProfiler	3.4.4

### Cell cultures

Mouse BV2 microglial cell line was obtained from Northeastern University, and cultured in high-glucose Dulbecco’s modified Eagle’s medium (DMEM) supplemented with 2% fetal bovine serum (FBS) 100 IU/ml penicillin, and 10 μg/ml streptomycin at 37 °C with an atmosphere of 5% CO_2_, routinely being tested for mycoplasma with Mycoplasma Stain Assay Kit (Beyotime). The cells were fasted with non-FBS DMEM media for 12–16 h before adding drugs. Then, BV2 cells were treated with LPS (500 ng/ml) for 12 h, followed by adding metformin (2.5 mM) or necrostatin-1 (Nec-1s, 500 nM, 2263, BioVision) for another 12 h.

### Cell viability analysis

CellTiter-Glo® Luminescent Cell Viability Assay (G7570, Promega) was performed to determine cell viability, as per the manufacturer’s instructions. Eight replicants were set for each treatment.

### Quantitative real-time polymerase chain reaction (qPCR)

The hypothalamus tissues or cells were collected, and total RNA was extracted with TRIzol reagent (Invitrogen, 15596026) following the manufacturer’s instructions. 500 ng of total RNA from each sample was reverse transcribed to cDNA with qPCR RT Master Mix with gDNA Remover (Toyobo, FSQ-301). According to the manufacturer’s instructions, qPCR was performed using SYBR Green Realtime Master Mix (Toyobo, QPK-201). Each quantitative reaction was performed in triplicate. To normalize the expression data, 18 s was used as the internal control gene. Relative gene expression levels were calculated using the 2-ΔΔCt method. The primer sequences used for the PCR amplification are presented in Table 3.

**Table 3:** Primer sequences for qPCR.

Primers		Sequences
Tnfa	Forward	5’-AGTCCGGGCAGGTCTACTTT-3’
	Reverse	5’-GGTCACTGTCCCAGCATCTT-3’
Il-1b	Forward	5’-CCCAACTGGTACATCAGCAC-3’
	Reverse	5’-TCTGCTCATTCACGAAAAGG-3’
Ccl2	Forward	5’-TGAATGTGAAGTTGACCCGT-3’
	Reverse	5’-AAGGCATCACAGTCCGAGTC-3’
Nfkb1	Forward	5’-ATGGCAGACGATGATCCCTAC-3’
	Reverse	5’-TGTTGACAGTGGTATTTCTGGTG-3’
Ripk1	Forward	5’-GACAGACCTAGACAGCGGAG-3’
	Reverse	5’-CCAGTAGCTTCACCACTCGAC-3’
Ripk3	Forward	5’-GGTGGTGCTACCAAGGAGTT-3’
	Reverse	5’-GAGATGGAAGACACGGCACT-3’
Mlkl	Forward	5’-TTAGGCCAGCTCATCTATGAACA-3’
	Reverse	5’-TGCACACGGTTTCCTAGACG-3’
18 s	Forward	5’-GACTCAACACGGGAAACCTC-3’
	Reverse	5’-TAACCAGACAAATCGCTCCAC-3’

### Immunofluorescence

For tissue immunofluorescence staining, the mice were anesthetized, and brains were obtained after heart perfusion with 4% paraformaldehyde (PFA, Sigma-Aldrich, 158127), followed by post-fixation in 4% PFA and dehydration through 20% and 30% sucrose orderly at 4 °C. Frozen sections (14-μm thickness) were blocked with QuickBlock™ Blocking Buffer (Beyotime Biotech) and incubated with primary antibodies overnight at 4 °C, including TNF-α (1:200, Abcam, AB-1793), Iba-1 (1:500, Wako, WFD-6884), TLR4 (1:100, Santa Cruz, SC-293072), and pMLKL (1:100, Sigma-Aldrich, MABC1158). Then, the primary antibodies were visualized with the appropriate secondary antibodies for 3 h incubation at room temperature with gentle rocking. After being washed with PBS, sections were mounted with 4,6-diamidino-2-phenylindole (DAPI) histology mounting medium (Sigma-Aldrich, F6057) and analyzed by Zeiss LSM 510 confocal microscope (Carl Zeiss).

For cell immunofluorescence staining, cells were seeded on cover glasses coated with Poly-l-lysine solution (Sigma-Aldrich, P8920 in a 24-well plate. After treatments indicated, the cells on cover glasses were fixed in fresh 4% PFA for 15 min at room temperature. Then, cells were rinsed with PBS, and the subsequential steps were the same with the procedures of tissue immunofluorescence staining.

### Immunoblotting

Total proteins of tissues or collected cells were obtained using Minute Total Protein Extraction Kit (SD-001, Invent Biotechnologies, Eden Prairie, MN, USA) supplemented with protease and phosphatase inhibitors (Roche) according to the instruction of the manufacture. Pierce BCA Protein Assay kits (Thermo Scientific, Rockford, USA) were used to determine protein concentrations. Thirty microgram proteins of each sample were separated on SDS-PAGE gels and then transferred to PVDF membranes, which were blocked with 5% non-fat milk for 1 h at room temperature. Membranes were incubated in the primary antibodies overnight at 4 °C as follows: IL-1β (1:1000, Abcam, AB9722), TLR4 (1:500), p-NF-κB (1:1000, Cell Signaling Technology, 3033), NF-κB (1:1000, Cell Signaling Technology, 8242), MLKL (1:1000, ProteinTech, 66675-1-Ig), RIPK3 (1:1000, ProteinTech, 17563-1-AP), RIPK1 (1:1000, BD Biosciences, 610459), HMGB1 (1:1000, Abcam, AB77302), HSP70 (1;1000, Cell Signaling Technology, 4872), and actin (1:5000, Cell Signaling Technology, 3077). Membranes were washed with TBS-T for 5 min three times the next day and then incubated with horseradish peroxidase-coupled secondary antibody for 1 h at room temperature. Signals were acquired using GE ImageQuant LAS4000, and quantification analysis was carried out by Image J software.

### Statistical analysis

All data were analyzed using the statistical software SPSS 19.0. Data are presented as the means ± standard error of the mean (SEM). Differences between the groups were analyzed by one-way or two-way ANOVA, followed by Tukey’s post hoc test for group comparisons. *p* < 0.05 was considered statically significant.

No methods were applied to determine whether the data met assumptions of the statistical approach used. Normal distributions were assumed.

## Supplementary information

Supplementary Table Legends

Supplementary Table 1 Excel spreadsheet listing DE genes in HFD vs. Chow

Supplementary Table 2 Excel spreadsheet listing DE genes in HM vs. HFD

## Data Availability

The data that support the findings of this study are available from the corresponding authors upon request.

## References

[1] Thaler JP, Yi CX, Schur EA, Guyenet SJ, Hwang BH, Dietrich MO (2012). Obesity is associated with hypothalamic injury in rodents and humans. J Clin Investig.

[2] Johnston AN, Wang Z (2020). HSP70 promotes MLKL polymerization and necroptosis. Mol Cell Oncol.

[3] Wang L, Chang X, Feng J, Yu J, Chen G (2019). TRADD mediates RIPK1-independent necroptosis induced by tumor necrosis factor. Front Cell Dev Biol.

[4] Huang Z, Zhou T, Sun X, Zheng Y, Cheng B, Li M (2018). Necroptosis in microglia contributes to neuroinflammation and retinal degeneration through TLR4 activation. Cell Death Differ.

[5] Caccamo A, Branca C, Piras IS, Ferreira E, Huentelman MJ, Liang WS (2017). Necroptosis activation in Alzheimer’sdisease. Nat. Neurosci.

[6] Horvath TL, Sarman B, Garcia-Caceres C, Enriori PJ, Sotonyi P, Shanabrough M (2010). Synaptic input organization of the melanocortin system predicts diet-induced hypothalamic reactive gliosis and obesity. Proc Natl Acad Sci USA.

[7] Valdearcos M, Robblee MM, Benjamin DI, Nomura DK, Xu AW, Koliwad SK (2014). Microglia dictate the impact of saturated fat consumption on hypothalamic inflammation and neuronal function. Cell Rep.

[8] Yerevanian A, Soukas AA (2019). Metformin: mechanisms in human obesity and weight loss. Curr Obes Rep.

[9] Wen S, Ling Y, Yang W, Shen J, Li C, Deng W (2017). Necroptosis is a key mediator of enterocytes loss in intestinal ischaemia/reperfusion injury. J Cell Mol Med.

[10] Luo T, Nocon A, Fry J, Sherban A, Rui X, Jiang B (2016). AMPK activation by metformin suppresses abnormal extracellular matrix remodeling in adipose tissue and ameliorates insulin resistance in obesity. Diabetes.

[11] Glueck CJ, Fontaine RN, Wang P, Subbiah MT, Weber K, Illig E (2001). Metformin reduces weight, centripetal obesity, insulin, leptin, and low-density lipoprotein cholesterol in nondiabetic, morbidly obese subjects with body mass index greater than 30. Metabolism.

[12] Jais A, Bruning JC (2017). Hypothalamic inflammation in obesity and metabolic disease. J Clin Investig.

[13] Thaler JP, Schwartz MW (2010). Minireview: Inflammation and obesity pathogenesis: the hypothalamus heats up. Endocrinology.

[14] Valdearcos M, Douglass JD, Robblee MM, Dorfman MD, Stifler DR, Bennett ML (2017). Microglial inflammatory signaling orchestrates the hypothalamic immune response to dietary excess and mediates obesity susceptibility. Cell Metab.

[15] Wang T, Perera ND, Chiam MDF, Cuic B, Wanniarachchillage N, Tomas D (2020). Necroptosis is dispensable for motor neuron degeneration in a mouse model of ALS. Cell Death Differ.

[16] Lloyd AF, Davies CL, Holloway RK, Labrak Y, Ireland G, Carradori D (2019). Central nervous system regeneration is driven by microglia necroptosis and repopulation. Nat Neurosci.

[17] Dionísio PEA, Oliveira SR, Amaral J, Rodrigues CMP (2019). Loss of microglial Parkin inhibits necroptosis and contributes to neuroinflammation. Mol Neurobiol.

[18] Chen AQ, Fang Z, Chen XL, Yang S, Zhou YF, Mao L (2019). Microglia-derived TNF-α mediates endothelial necroptosis aggravating blood brain-barrier disruption after ischemic stroke. Cell Death Dis.

[19] Keshk WA, Ibrahim MA, Shalaby SM, Zalat ZA, Elseady WS, Xu H, et al. Redox status, inflammation, necroptosis and inflammasome as indispensable contributors to high fat diet (HFD)-induced neurodegeneration; Effect of N-acetylcysteine (NAC). The pseudokinase MLKL regulates hepatic insulin sensitivity independently of inflammation. TLR9 is up-regulated in human and murine NASH: pivotal role in inflammatory recruitment and cell survival. (1096-0384 (Electronic)).

[20] Yu M, Huang H, Dong S, Sha H, Wei W, Liu C (2019). High mobility group box-1 mediates hippocampal inflammation and contributes to cognitive deficits in high-fat high-fructose diet-induced obese rats. Brain Behav Immun.

[21] Kalsbeek MJ, Wolff SE, Korpel NL, la Fleur SE, Romijn JA, Fliers E, et al. The impact of antidiabetic treatment on human hypothalamic infundibular neurons and microglia. JCI Insight 2020;5. 10.1172/jci.insight.133868 PMC7455135 32814716

[22] Kodali M, Attaluri S, Madhu LN, Shuai B, Upadhya R, Gonzalez JJ (2021). Metformin treatment in late middle age improves cognitive function with alleviation of microglial activation and enhancement of autophagy in the hippocampus. Aging Cell.

[23] Docrat TF, Nagiah S, Chuturgoon AA (2021). Metformin protects against neuroinflammation through integrated mechanisms of miR-141 and the NF-kB-mediated inflammasome pathway in a diabetic mouse model. Eur J Pharm.

[24] Lv W-S, Wen J-P, Li L, Sun R-X, Wang J, Xian Y-X (2012). The effect of metformin on food intake and its potential role in hypothalamic regulation in obese diabetic rats. Brain Res.

[25] Fernandez-Lizarbe S, Montesinos J, Guerri C (2013). Ethanol induces TLR4/TLR2 association, triggering an inflammatory response in microglial cells. J Neurochem.

[26] He S, Liang Y, Shao F, Wang X (2011). Toll-like receptors activate programmed necrosis in macrophages through a receptor-interacting kinase-3-mediated pathway. Proc Natl Acad Sci USA.

[27] Jing Y, Wu F, Li D, Yang L, Li Q, Li R (2018). Metformin improves obesity-associated inflammation by altering macrophages polarization. Mol Cell Endocrinol.

[28] Silke J, Rickard JA, Gerlic M (2015). The diverse role of RIP kinases in necroptosis and inflammation. Nat Immunol.

[29] Kearney CJ, Cullen SP, Clancy D, Martin SJ (2014). RIPK1 can function as an inhibitor rather than an initiator of RIPK3-dependent necroptosis. FEBS J.

[30] Najafov A, Luu HS, Mookhtiar AK, Mifflin L, Xia HG, Amin PP (2021). RIPK1 promotes energy sensing by the mTORC1 pathway. Mol Cell.

[31] Lee K-H, Kang T-B (2019). The molecular links between cell death and inflammasome. Cells.

