# Supplementary material for: Metformin attenuates hypothalamic inflammation via downregulation of RIPK1-independent microglial necroptosis in diet-induced obese mice
Source: Cell Death Discov. 2021 Nov 8;7:338. doi: 10.1038/s41420-021-00732-5 (PMC8575871; doi:10.1038/s41420-021-00732-5)
Supplement: Supplementary file 1 — Supplementary Table Legends [file 41420_2021_732_MOESM1_ESM.docx]

Supplementary Table 1: Excel spreadsheet listing DE genes in HFD vs. Chow.

Supplementary Table 2: Excel spreadsheet listing DE genes in HM vs. HFD.
